# Profile of Clinical and Analytical Parameters in Bronchiectasis Patients during the COVID-19 Pandemic: A One-Year Follow-Up Pilot Study

**DOI:** 10.3390/jcm11061727

**Published:** 2022-03-21

**Authors:** Liyun Qin, Filipe Gonçalves-Carvalho, Yingchen Xia, Jianhua Zha, Mireia Admetlló, José María Maiques, Sandra Esteban-Cucó, Xavier Duran, Alicia Marín, Esther Barreiro

**Affiliations:** 1 Muscle Wasting and Cachexia in Chronic Respiratory Diseases and Lung Cancer Research Group, Pulmonology Department, IMIM-Hospital del Mar, Parc de Salut Mar, Parc de Recerca Biomèdica de Barcelona (PRBB), 08003 Barcelona, Spain; liyun.qin@e-campus.uab.cat (L.Q.); 361439919013@email.ncu.edu.cn (Y.X.); 361439918044@email.ncu.edu.cn (J.Z.); madmetllo@parcdesalutmar.cat (M.A.); 2 Department of Medicine, Universitat Autònoma de Barcelona, 08035 Barcelona, Spain; 3 Pulmonology Department, Hospital Germans Trias i Pujol, 08916 Badalona, Spain; filipegscarvalho90@gmail.com (F.G.-C.); amarin.germanstrias@gencat.cat (A.M.); 4 Centro de Investigación en Red de Enfermedades Respiratoria (CIBERES), Instituto de Salud Carlos III (ISCIII), 08003 Barcelona, Spain; 5 Department of Thoracic Surgery, The First Affiliated Hospital of Nanchang University, Nanchang 330209, China; 6 Radiology Department, Imatge Mèdica Intercentres-Parc de Salut Mar, Hospital del Mar, 08003 Barcelona, Spain; jmaiques@psmar.cat; 7 Laboratori de Referència de Catalunya, Clinical Microbiology and Parasitology Department, 08820 Barcelona, Spain; sestebanc@lrc.cat; 8 Scientific and Technical Department, Hospital del Mar-IMIM, 08003 Barcelona, Spain; xduran@imim.es; 9 Department of Medicine and Life Sciences (MELIS), Universitat Pompeu Fabra (UPF), 08002 Barcelona, Spain

**Keywords:** non-cystic fibrosis bronchiectasis, systemic inflammation and immunoglobulins, nutritional status, lung function, severity scores, immunoglobulins, one-year follow-up

## Abstract

Whether the COVID-19 pandemic may have modified the clinical planning and course in bronchiectasis patients remains to be fully elucidated. We hypothesized that the COVID-19 pandemic may have influenced the management and clinical outcomes of bronchiectasis patients who were followed up for 12 months. In bronchiectasis patients (n = 30, 23 females, 66 years), lung function testing, disease severity [FEV_1_, age, colonization, radiological extension, dyspnea (FACED), exacerbation (EFACED)] and dyspnea scores, exacerbation numbers and hospitalizations, body composition, sputum microbiology, and blood analytical biomarkers were determined at baseline and after a one-year follow-up. Compared to baseline (n = 27, three patients dropped out), in bronchiectasis patients, a significant increase in FACED and EFACED scores, number of exacerbations, and erythrocyte sedimentation rate (ESR) was observed, while FEV_1_, ceruloplasmin, IgE, IgG, IgG aspergillus, IgM, and IgA significantly decreased. Patients presenting colonization by *Pseudomonas aeruginosa* (PA) remained unchanged (27%) during follow-up. In bronchiectasis patients, FEV_1_ declined only after a one-year follow-up along with increased exacerbation numbers and disease severity scores, but not hospitalizations. However, a significant decrease in acute phase-reactants and immunoglobulins was observed at the one-year follow-up compared to baseline. Despite the relatively small cohort, the reported findings suggest that lung function impairment may not rely entirely on the patients’ inflammatory status.

## 1. Introduction

Bronchiectasis is a chronic respiratory condition characterized by abnormalities in the airways of the patients that lead to increased sputum production, cough, chest pain, and eventually dyspnea. The etiology varies widely from previous lung infections to genetic diseases that are diagnosed early in childhood [1,2,3]. Moreover, other chronic respiratory diseases, namely chronic obstructive pulmonary disease (COPD) and asthma, may be associated with bronchiectasis [1,4,5,6,7]. The prevalence of bronchiectasis is progressively increasing as the diagnostic tools become more widely available in different clinical settings [1,8,9,10]. Patients with bronchiectasis are prone to suffer acute exacerbations, which in many cases may require hospitalizations [11,12,13].

In patients with chronic airway diseases, follow-up studies are of interest to monitor the potential loss of respiratory function and control of symptoms. An eight-year follow-up investigation [14] concluded that female patients were predominant, had persistent symptoms, and a severe loss of lung function was detected. The same investigators also demonstrated [15] that patients with bronchiectasis were colonized by the same bacterium over a five-year follow-up period, and that phenotypic features were associated with different pathogens. A more recent investigation [16] demonstrated that multimorbidity was common in patients with bronchiectasis and negatively influenced survival. Furthermore, the line was also put forward that specific disease scores helped predict mortality and outcomes during the five-year follow-up period in the same study [16]. Whether similar findings can be observed in follow-up studies of shorter duration remains to be studied.

The new human pathogen known as the Severe Acute Respiratory Syndrome Coronavirus 2 (SARS-CoV-2) was identified for the first time in the province of Wuhan (China) in December 2019 [17,18]. SARS-CoV-2 alters several organs, among which the lungs are the most commonly and severely affected. The coronavirus disease (COVID)-19 has caused an unprecedented pandemic worldwide that started in 2020 [19]. Lifestyle and personal habits have been modified as a result of the widespread pandemic, with strong implications in the management of patients, particularly of those with chronic respiratory diseases. Whether the COVID-19 pandemic has affected the habitual management planning and monitoring of the outpatient clinics in bronchiectasis deserves to be investigated.

On the basis of this, we hypothesized that the COVID-19 pandemic may have influenced the management planning and clinical outcomes of patients with bronchiectasis who were followed up for at least 12 consecutive months in a specialized outpatient clinic. Thus, our objectives were to assess during the COVID-19 pandemic (the year 2020) in patients with non-cystic fibrosis bronchiectasis that were consecutively recruited and followed up for at least 12 months the following clinical outcomes: (1) airway obstruction, disease severity scores, the number of acute exacerbations, and COVID-19 episodes, if any (2) nutritional and inflammatory parameters, and (3) systemic immunoglobulins. All the measurements were conducted at baseline and at the end of the study period in the year 2021.

## 2. Methods

This was a prospective, follow-up investigation in which 30 patients (7 males) were recruited consecutively from the Multidisciplinary Bronchiectasis Unit of the Respiratory Department at Hospital del Mar (Barcelona, Spain) from 9 July 2019 to 10 March 2020. Twenty-five patients were recruited in the months of July, September, October, November, and December 2019, while five patients were recruited in March 2020. The 25 patients recruited in 2019 were followed up in 2020 up until April 2021 with several month-delays due to the pandemic period. In the first half of 2020, outpatient consultations were cancelled in order to assist all the hospitalized COVID-19 patients, especially those requiring ventilatory support (either non-invasive or invasive). Patients recruited in March 2020 were followed up until May 2021. Thus, all the participants were followed-up during the year 2020 (a period at which the pandemic was more pervasive in our societies). Two patients had concomitant COVID-19 during the follow-up period: in December of the year 2020 and in January of 2021. However, they did not experience any acute bronchiectasis exacerbation as a result of COVID-19.

All the patients participated in three consultations: baseline, 6-month interim, and 12-month visit. Twenty-seven patients (3 patients dropped out, two from 2019 and one from 2020 recruitment periods) were followed up for one year (range from 12 to 18 months) last follow-up visit completed on 4 May 2021). Inclusion criteria were as follows: adults (18 years and over), diagnosis of non-cystic fibrosis (CF) bronchiectasis by high-resolution computerized tomography (HRCT) [1,20], and no previous exacerbations of the disease at least 4 weeks prior to study entry. Exclusion criteria for all the patients included other chronic cardiovascular or respiratory disorders, acute and chronic respiratory failure, chronic metabolic diseases, signs of severe bronchial inflammation and/or infection, current or recent invasive mechanical ventilation, long-term oxygen therapy, and poor collaboration. The majority of the patients recruited for the purpose of the investigation had a mild-to-moderate disease severity according to lung function impairment [21], disease severity scores, and radiological extension [1,22,23,24,25]. All the patients were stable: no acute exacerbations in the last four weeks prior to study entry. Approval was obtained from the institutional Ethics Committee on Human Investigation (Hospital del Mar-IMIM, Barcelona, Spain, protocol # 2019/8482/1, 14 March 2019) following the World Medical Association guidelines (Declaration of Helsinki, Fortaleza, Brazil, October 2013) for research on human beings. Informed written consent was obtained from all participants.

### 2.1. Clinical Assessment at Baseline and at One-Year Follow-Up Time-Points

Body weight and height were measured after a fasting period of at least four hours in all the patients. Moreover, blood samples were also obtained from the arm vein after an overnight fasting period.

The following clinical variables were obtained at baseline and at one-year follow-up time-points: body mass index (BMI), lung function parameters, exercise capacity, dyspnea, number of acute exacerbations/patient/year, hospitalizations for acute exacerbations/patient/year, nutritional status, therapeutic strategies, and systemic inflammatory parameters. Lung function parameters were determined in all study subjects following standard procedures and reference values commonly used in our laboratory [26,27,28,29,30]. Exercise capacity was assessed through the six-minute walking distance following previous methodologies [31]. In order to prevent unnecessary irradiation of the patients, HRCT was only carried out at baseline.

### 2.2. Bronchiectasis Disease Severity Scores

The FACED score [24] was used for clinical estimation of the patients’ status by incorporating variables: forced expiratory volume in one second (FEV_1_) [F; cutoff, 50%; 0 or 2 points], age [A; cutoff, 70 years; 0 or 2 points], chronic colonization by *Pseudomonas aeruginosa* [C; yes, 0 or 1 point], radiological extension [E; number of lobes affected; cutoff, two lobes; 0 or 1 point], and dyspnea [D; cutoff, grade 2 on the modified Medical Research Council (mMRC) dyspnea scale; 0 or 1 point]). Severity classification according to FACED scores was as follows: (1) 0–2, mild disease, (2) 3–4, moderate disease, and (3) 5–7, severe disease.

The EFACED score represents [25] (E: exacerbations with hospitalization in the previous year; F: FEV_1_; A: age; C: chronic colonization by *Pseudomonas aeruginosa*; E: radiological extension [number of pulmonary lobes affected]; and D: dyspnea). Severity classification according to EFACED scores was as follows: (1) 0–3, mild disease, (2) 4–6, moderate disease, and (3) 7–9, severe disease.

The bronchiectasis severity index (BSI) score [22] (age [maximum value: 6 points], BMI [maximum value: 2 points], FEV_1_ [maximum value: 3 points], hospital admission prior to study [maximum value: 5 points], exacerbations prior to the study [maximum value: 2 points], Medical Research Council (MRC) dyspnea scale [maximum value: 3 points], chronic colonization by *Pseudomonas aeruginosa* (PA) [maximum value: 3 points], chronic colonization by other microorganisms [maximum value: 1 points], radiological extension [maximum value: 1 points]). Severity classification according to BSI scores was as follows: (1) 0–4, mild disease, (2) 5–8, moderate disease, and (3) ≥9, severe disease.

### 2.3. Radiological Features and Extension

High-resolution computer tomography (HRCT)-scans were used to evaluate the radiological extension of the bronchiectasis in all the study patients only at baseline. Scores for each patient were calculated by two independent observers following previously established criteria [32,33]. The extent of bronchiectasis (ES) was scored for each lobe as follows: grade 0 = no disease; grade 1 = one or partial bronchopulmonary segment involved; grade 2 = two or more bronchopulmonary segments involved. The lingula was considered a separate lobe in this analysis. The bronchial dilatation (DS) was quantified relative to the adjacent pulmonary arteries as follows: grade 0 = no bronchiectasis; grade 1 = less than twice (200%) diameter of adjacent pulmonary artery (APA); grade 2 = 200–300% diameter of APA; grade 3 = >300% diameter of APA. Bronchial wall thickness (TS) was scored as follows: grade 0 = none; grade 1 = 50% of APA, grade 2 = 50–100% of APA; grade 3 = >100% of APA.

Global scores of both lungs were taken for extension, bronchial dilatation and bronchial wall thickness. The total extent of bronchiectasis (TES) was taken as the sum of the ES for each of the six lobes. The global severity of bronchial dilatation (GDS) was as the “sum of the extent score multiplied by the dilatation score for each lobe”, divided by the “total extent score” (GDS = ∑(ES × DS)_1–6_/TES). Similarly, the global severity of bronchial wall thickness (GWTS) was estimated as the “sum of the extent score multiplied by the thickness score for each lobe” divided by the “total extent score” (GTS = ∑(ES × TS)_1–6_/TES).

### 2.4. Microbiological Diagnosis

Spontaneous or induced sputum samples were obtained from all the patients. Sputum samples were analyzed in the microbiology laboratory. Conventional semi-qualitative bacterial and fungal cultures were performed. An initial Gram staining was performed in all the samples prior to culturing the sputum according to the Murray and Washington criteria [34] (Table 1).

Sputum samples were cultured in Agar Chocolate, Agar Columbia Nalidixic Acid (CNA), Agar MacConkey, and Agar Sabouraud. Bacterial cultures were read at 24 h and 48 h time-points, while those of fungal cultures were read every 24 h for five consecutive days. Antibiotic sensitivity was tested using the microdilution method or disc diffusion following the regulations of the European Committee on Antimicrobial Susceptibility Testing (EUCAST) [35]. The strains were frozen in two separate freezing tubes at −80 °C.

When available, mycobacteria cultures were also performed in the patients. Upon sample decontamination using the sodium hydroxide (NaOH) method, samples were cultured in a solid medium culture of Lowenstein-Jensen Media (BD BLL^TM^) and a liquid medium BACT/ALERT^®^ (BioMerieux, SA F-69280 Marcy I’Etoile, Lyon, France) or BACTEC™ MGIT™ 960 (BD) for 40 consecutive days.

### 2.5. Statistical Analysis

The normality of the study variables was tested using the Shapiro–Wilk test. Data are expressed as mean and standard deviation (SD) in tables and figures. A post-hoc power was calculated on the basis of the parameters IgE and IgG for the Paired Sample *t*-Test applied to check differences from baseline to follow-up measurements. The post-hoc power calculation was 82.94% for the sample size estimated in the study. At baseline, patients were also analyzed separately according to the presence or absence of colonization by PA. Potential differences of quantitative variables between these two groups were assessed using the Student’s *t*-test or Mann-Whitney U test for parametric and non-parametric distributions, respectively. A Chi-square test was used to assess potential differences in categorical variables between the PA colonization group and the non-PA colonization group. Potential differences of quantitative variables between the baseline and one-year follow-up time-points were explored using the Paired Samples *t*-Test or Two-Related Samples Tests for parametric and non-parametric distributions. All statistical analyses were performed using the software SPSS 23.0 (SPSS Inc., Chicago, IL, USA). Statistical significance was established at *p* ≤ 0.05.

## 3. Results

### 3.1. General Characteristics at Baseline

Table 2 illustrates the baseline clinical characteristics of all the patients. Bronchiectasis severity was classified as mild-to-moderate according to FACED, EFACED, and BSI scores (Table 2). Patients predominantly showed a mild-to-moderate airway obstruction (Table 2). Specifically, 15 patients had an FEV_1_ predicted greater than 80% (81–124% range), 11 patients had an FEV_1_ predicted comprised between 50% and 80% (50–73% range), and four patients had an FEV_1_ predicted lower than 50% (37–48% range).

Most patients were females, and post-infectious sequelae were the most frequent etiological factor in this series (Table 2). At baseline, patients colonized by PA were significantly younger, the walking distance was greater, while the mMRC score was significantly lower than in non-PA patients (Table 2). At baseline, no significant differences were observed in the study nutritional parameters between PA- and non-PA groups of patients (Table 2). PA-colonized patients exhibited a significantly greater extension of their bronchiectasis, as indicated by the total extension and global scores (Table 2). No significant differences in the number of acute exacerbations/patient/year or hospitalizations/patient/year due to exacerbations were seen between PA and non-PA groups of patients in this cohort (Table 2).

Therapy details are also described in Table 2 below. During follow-up, patients with PA followed an eradication protocol based on the use of full doses of quinolones or co-trimoxazole (Stenotrophomonas maltophilia) for at least three consecutive weeks as established in the Spanish guidelines for non-severe bronchiectasis [36].

### 3.2. Systemic Inflammatory Parameters and Immunoglobulins (Ig) at Baseline

Levels of the systemic inflammatory parameters at baseline for all the patients are shown in Table 3. At baseline, patients with PA colonization showed greater levels of IgG, IgM, and IgA than patients with non-PA colonization (Table 3).

### 3.3. General Clinical Characteristics at One-Year Follow-Up

**Figure 1:**
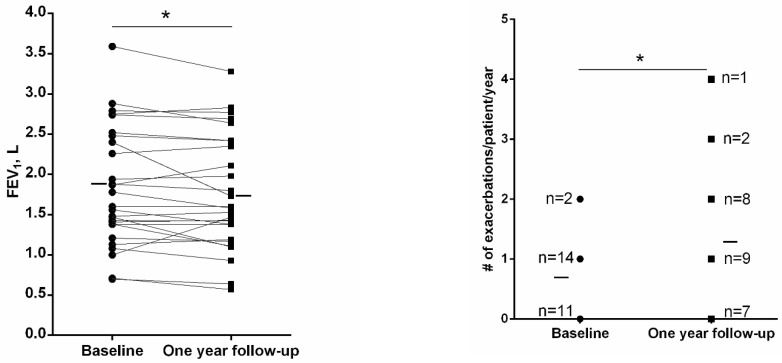
Individual and mean values of FEV_1_ lung function parameter and the number of exacerbations/patient/year during the 12-month follow-up period at baseline and at one-year follow-up in bronchiectasis patients. Twenty patients had at least one exacerbation during follow-up. Abbreviations: n, number. Statistical significance is as follows: * *p* ≤ 0.05 comparisons between baseline and one-year follow-up time-points.

Three patients dropped out (two patients recruited in 2019 and one recruited in 2020) from the one-year follow-up part of the study for personal reasons; thus, 27 patients were followed up for 12 months. Compared to baseline, at one-year follow-up, a significant reduction in FEV_1_ (70 mL) was observed in the study patients, while the number of exacerbations significantly increased (88%, Figure 1). Indeed, 20 out of 30 patients had at least one exacerbation during the 12-month follow-up period, and 15 patients showed an increased number of exacerbations compared to baseline (Table 4). No significant differences were seen in the rate of FEV_1_ decline (either absolute and % predicted values) at the end of the study period between patients with an increased number of exacerbations during follow-up and those without, n = 20 and n = 7, *p* = 0.893 and *p* = 0.912, respectively). Furthermore, the rate of FEV_1_ decline (either absolute and % predicted values) at 12-month time-point did not significantly differ between patients with a former history of frequent exacerbations (two or more in the year prior to study entry) and those without (n = 5 and n = 25, *p* = 0.835, respectively).

**Figure 2:**
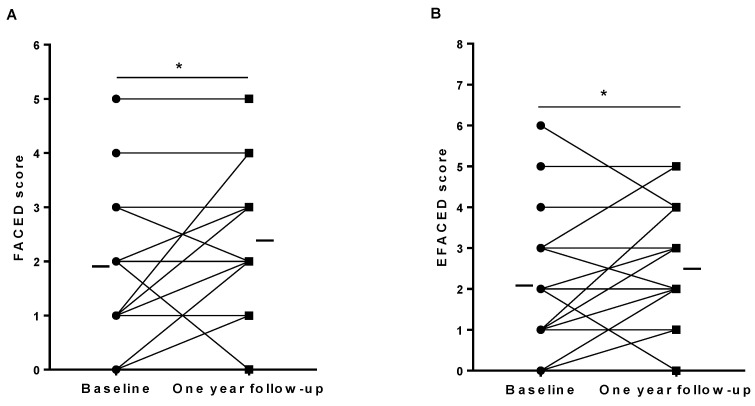
Individual and mean values of (**A**) FACED score and (**B**) EFACED score at baseline and at one-year follow-up in bronchiectasis patients. Statistical significance is as follows: * *p* ≤ 0.05 comparisons between baseline and one-year follow-up time-points.

No significant differences were observed between one-year follow-up and baseline time-points in other lung function parameters or exercise capacity (Table 5). No significant differences were observed between the one-year follow-up and baseline time-points of the study nutritional parameters except for hemoglobin, which significantly declined (Table 5). The disease severity scores FACED and EFACED, but not BSI, significantly increased after the one-year follow-up compared to baseline (Figure 2A,B and Table 5, respectively). The rate of FEV_1_ decline (absolute and % predicted values) did not show any significant differences between patients with and without a rise in the bronchiectasis severity scores (FACED n = 9 and n = 18, EFACED n = 10 and n = 17, and BSI n = 10 and n = 17, *p* = 0.899, *p* = 0.493, and *p* = 0.619, respectively).

### 3.4. Systemic Inflammatory Parameters and Immunoglobulins (Ig) at One-Year Follow-Up

**Figure 3:**
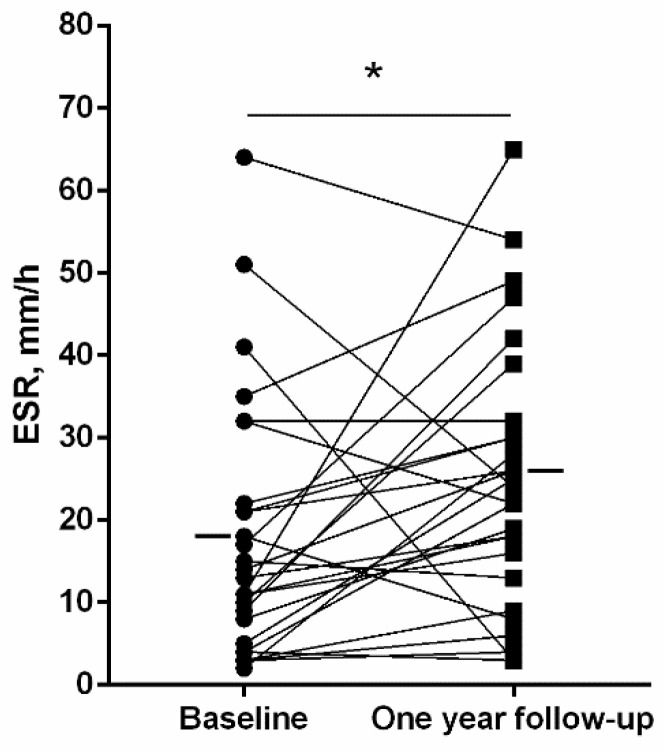
Individual and mean values of levels of ESR at baseline and at one-year follow-up in bronchiectasis patients. Statistical significance is as follows: * *p* ≤ 0.05 comparisons between baseline and one-year follow-up time-points.

**Figure 4:**
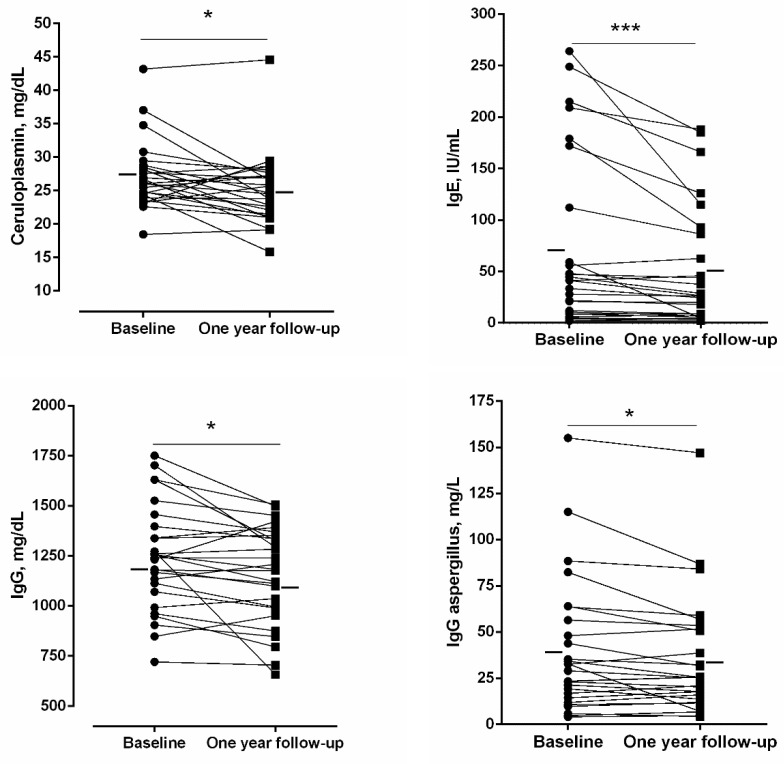
Individual and mean values of levels of ceruloplasmin, IgE, IgG and IgG aspergillus at baseline and at one-year follow-up in bronchiectasis patients. Statistical significance is as follows: * *p* ≤ 0.05, *** *p* ≤ 0.001 comparisons between baseline and one-year follow-up time-points.

**Figure 5:**
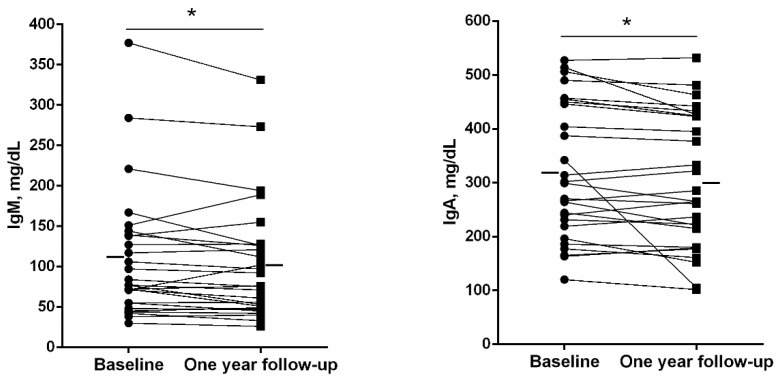
Individual and mean values of levels of IgM and IgA at baseline and at one-year follow-up in bronchiectasis patients. Statistical significance is as follows: * *p* ≤ 0.05 comparisons between baseline and one-year follow-up time-points.

A significant rise in the erythrocyte sedimentation rate (ESR) was detected in the patients at one-year follow-up compared to baseline (Figure 3), whereas no differences were observed in the number of leukocytes, neutrophils, lymphocytes, or eosinophils (Table 6). The number of platelets, however, significantly declined at one-year follow-up compared to baseline (Table 6). Blood levels of ceruloplasmin, IgE, IgG, IgG aspergillus, IgM, and IgA significantly declined at one-year follow-up compared to baseline in the bronchiectasis patients (Figure 4 and Figure 5, respectively).

### 3.5. Microbiological Status of the Sputum at Baseline and One-Year Follow-Up

Table 7 illustrates the microbiological results from the sputum cultures of all the patients at baseline and at one-year follow-up. No significant differences were observed in FEV_1_ decline (absolute and % predicted values), disease severity scores, or the number of exacerbations/patient/year between patients showing newly acquired colonization and those without (*p* > 0.05 all analyses).

## 4. Discussion

In the current investigation, the most relevant findings were that outpatients with bronchiectasis consecutively recruited from a specialized clinic during the COVID-19 pandemic were predominantly females, exhibited mild airway obstruction and disease severity as indicated by specific scores, and post-infectious was the commonest etiology in this series of patients. A relatively normal follow-up period was attained despite the pandemic, in which all the participants were followed up for a minimum of 12 months, except for short delays in the consultations as a result of the heavy burden of COVID-19 patients in the first half of the year 2020 and the subsequent reorganization of the clinics that followed. The follow-up period consisted of three different appointments: baseline, six-month, and 12-month visits. Importantly, at one-year follow-up compared to baseline, the patients showed a significant reduction in FEV_1_ (70 mL), along with a significant rise in the number of exacerbations per patient, disease severity scores, and in levels of ESR parameter. Nonetheless, levels of the inflammatory parameters ceruloplasmin and the study immunoglobulins were significantly reduced in all the patients at one-year follow-up compared to baseline. These are relevant results that are further discussed below.

A major relevant finding in this study was the significant decline in absolute values of FEV_1_ observed among the bronchiectasis patients right after only one-year follow-up with respect to baseline measurements. The 70-mL loss of FEV_1_ detected in the patients in the 12-month visit is far greater than that reported to happen under physiological conditions in normal subjects or even in smokers [37]. In this cohort, a loss of 52 mL was calculated in the year prior to study entry. Importantly, an association between the degree of radiological emphysema and small airway disease and FEV_1_ decline was also demonstrated in COPD patients, particularly in those with mild-to-moderate disease [38]. In the present study, the 70-mL decline in FEV_1_ values at one-year follow-up can only be attributable to the presence of bronchiectasis since none of the patients smoked. In addition, only one patient also had concomitant COPD, and this patient dropped out from the study at month seven. Thus, bronchiectasis per se elicited a significant decline in lung function as early as 12 months of the follow-up period in patients aged 60 to 70 years old. As the loss in lung function was greater under the study period (70 mL) than that observed in the year prior to study entry (52 mL), the mechanisms whereby FEV_1_ decline progressively increases in bronchiectasis patients should be fully understood in future investigations.

The number of acute exacerbations, but not those of hospitalizations, increased in the patients at one-year follow-up compared to baseline. In fact, 20 patients experienced an exacerbation during the follow-up period, and 15 out the 30 patients had an increase in the number of acute exacerbations during the 12-month follow-up period. Indeed, acute exacerbations are common in patients with bronchiectasis [11,12,13,39], which in certain cases may require hospitalizations. Recently, it has been reported that a history of previous hospitalizations, heart failure, and high disease severity scores were associated with a greater risk for hospitalizations in patients with acute exacerbations of bronchiectasis [13]. Furthermore, in the present investigation, FACED and EFACED scores were also significantly greater at one-year follow-up than at baseline in the bronchiectasis patients. Whether disease severity scores may help predict the risk of acute exacerbations and/or hospitalizations in patients with bronchiectasis remains to be fully elucidated. In line with this, it has been proposed that FACED and BSI may not be all that helpful to predict the risk of exacerbations in patients with bronchiectasis [40]. Nevertheless, BSI and FACED scores were very useful markers to predict the five-year mortality in bronchiectasis patients [41].

Interestingly, levels of the parameter ESR were significantly greater after the one-year follow-up than baseline levels in the study patients. ESR is a systemic inflammatory marker that can be used as a prognostic tool in clinical settings of other respiratory diseases [42]. In stable bronchiectasis patients, ESR values did not significantly correlate with disease severity in a retrospective study [43]. Whether ESR may have a prognostic value in bronchiectasis patients warrants further attention.

Ceruloplasmin is a protein synthesized in the liver that carries copper and is also involved in iron metabolism. As an acute phase-reactant, ceruloplasmin levels rise in inflammatory processes [44,45]. In the present study, a small but significant decline (from 27 to 25 mg/dL) in ceruloplasmin levels was observed in bronchiectasis patients at one-year follow-up compared to baseline. The precise biological mechanisms whereby this inflammatory parameter may be involved in the pathophysiology of bronchiectasis need to be explored in future investigations.

Immunoglobulins, along with B and T cells, are major components of the adaptive immune response in humans. In the current investigation, baseline values of the analyzed immunoglobulins were within the normal range for all the patients. Importantly, at one-year follow-up, the serum values of IgE, IgG, IgG aspergillus, IgM, and IgA significantly declined compared to baseline levels. As indoor and outdoor pollution are potential stimuli of the production of acute phase-reactants and immunoglobulins [46], it is likely that the lockdowns and the reduced outdoor activity experienced during the COVID-19 pandemic may have substantially contributed to the decline in the levels of these markers in the study population. Whether or not the reduction in immunoglobulins and acute phase-reactant levels may influence long-term clinical outcomes and disease prognosis warrants further attention in future investigations.

### Study Critique

A potential criticism is the use of a relatively small cohort that has been analyzed in the study. However, outpatients were consecutively recruited in a specialized clinic on the basis of very strict inclusion and exclusion criteria. Moreover, a power calculation was almost 83% in this specific cohort, endorsing the reliability of the study results. Moreover, all the patients were followed up during the COVID-19 pandemic in 2020, when several lockdowns and curfews were applied in society. Despite these measures, non-COVID-19 respiratory outpatients were equally attended in our specialized clinic, and the established visits were highly respected as described in Methods (every six months). All the patients maintained social distancing and were wearing masks at all times (indoors and outdoors) as these measurements were enforced by the local authorities during the pandemic. Only two patients out of 30 had mild COVID-19, without leading to acute exacerbations during follow-up. These results suggest that these preventive measurements safeguarded patients from developing COVID-19.

Patients with PA infection at baseline were significantly younger than those with no PA infection, and the mMRC score was smaller, probably due to the age factor. Moreover, at baseline, patients with PA infection exhibited greater levels of immunoglobulins and larger radiological extension in the HRCT than non-PA patients, while showing no differences in the number of acute exacerbations or hospitalizations in the previous year. Despite the relevance and interest of these findings, caution should be taken as the number of patients in the PA-infected group was relatively small.

Another potential limitation might be the drop-out of three patients during the follow-up period of the study. Nonetheless, the statistical power (83%) was sufficiently high to ensure the validity of the results on the basis of the 27 patients who participated both at baseline and during follow-up. It should also be mentioned that only two patients out of 27 had mild COVID-19 in late 2020 and early 2021, respectively. None of the patients had been vaccinated over the study period, as vaccines against SARS-CoV-2 were not vastly available at that time.

## 5. Conclusions

In patients with bronchiectasis, a significant decline in FEV_1_ was observed only after the one-year follow-up period, along with a rise in the number of acute exacerbations and disease severity scores, but not of hospitalizations. However, a significant decrease in acute phase-reactants and immunoglobulins was observed at one-year follow-up compared to baseline. These findings suggest that the lung function impairment seen in these patients, particularly of the airways, may not rely entirely on their systemic inflammatory status. Identification of the pathophysiological mechanisms leading to substantial lung function impairment in bronchiectasis patients warrants further attention in future research. Despite the relatively small cohort analyzed in this study, the reported findings have clear clinical implications in the management of patients with bronchiectasis.

## Figures and Tables

**Table 1:** Criteria for evaluation of the quality of sputum specimens.

Score	Epithelial Cells	Leukocytes	Quality	Culture
1	>25	<10	Very poor	No
2	>25	18–25	Poor	No
3	>25	>25	Dubious	Yes
4	18–25	>25	Sufficient	Yes
5	<10	>25	Good	Yes
6	<25	<25	Uncertain	Yes

Adapted from Murray P.R. et al. See reference [34].

**Table 2:** Baseline general characteristics of all bronchiectasis patients, and of the two groups of patients.

$\mathrm{Anthropometry}\;\bar{x}\;(\mathrm{SD})$	All Patients N = 30	Non-*Pseudomonas aeruginosa* N = 21	*Pseudomonas aeruginosa* N = 9
Age, years	66 (12)	69 (11)	61 (12) *
Female, N/male, N	23/7	18/3	5/4
Body weight, kg	64 (16)	63 (18)	66 (11)
Height, cm	158 (10)	157 (11)	162 (8)
BMI (kg/m^2^)	25 (4)	25.5 (4.9)	24.9 (2.8)
Etiology			
Post-infectious, N (%)	22 (73)	14 (67)	8 (89)
COPD, N (%)	1 (3)	1 (4)	0 (0)
Unknown etiology, N (%)	7 (24)	6 (29)	1 (11)
$\mathrm{Lung}\;\mathrm{function}\;\mathrm{and}\;\mathrm{exercise}\;\mathrm{capacity},\;\bar{x}\;($SD)			
FEV1, L	1.78 (0.71)	1.76 (0.71)	1.84 (0.75)
FEV1, % predicted	76 (25)	80 (25)	65 (21)
FVC, L	2.68 (0.92)	2.52 (0.78)	3.05 (1.15)
FVC, % predicted	85 (18)	87.9 (18.5)	78.9 (16.5)
FEV1/FVC	68 (11)	68.6 (11.2)	65.6 (12.3)
6-min walking distance, meters	473 (96)	450 (95)	534 (71) *
Distance, % predicted	98 (17)	96 (16)	105 (19)
Smoking history			
Ex-smokers, N (%)	9 (30)	6 (29)	3 (33)
Never smokers, N (%)	21 (70)	15 (71)	6 (67)
Packs-year,	22 (15)	23 (18)	12 (3)
Disease severity			
FACED score,	1.9 (1.3)	1.95 (1.43)	1.89 (1.05)
Mild, N	20	13	7
Moderate, N	8	6	2
Severe, N	2	2	0
EFACED score,	2.1 (1.5)	2.05 (1.50)	2.33 (1.73)
Mild, N	25	18	7
Moderate, N	5	3	2
Severe, N	0	0	0
$\mathrm{BSI}\;\mathrm{score},\;\bar{x}\;($SD)	5.5 (3.2)	5.19 (2.62)	6.33 (4.36)
Mild, N	14	10	4
Moderate, N	11	8	3
Severe, N	5	3	2
$\mathrm{mMRC}\;\mathrm{score},\;\bar{x}\;($SD)	0.63 (0.67)	0.71 (0.46)	0.22 (0.44) *
# exacerbations/patient/year,	0.87 (1.00)	0.86 (0.66)	0.89 (1.62)
# hospitalizations/patient for exacerbations in the previous year,	0.10 (0.31)	0.05 (0.22)	0.22 (0.44)
$\mathrm{Nutritional}\;\mathrm{assessment},\;\bar{x}\;($SD)			
Hemoglobin, g/dL	13.9 (11.1)	13.7 (1.1)	14.3 (1.2)
Hematocrit, %	42.0 (3.7)	41.4 (3.6)	43.4 (3.8)
Glucose, mg/dL	94.4 (25.5)	98.8 (29.1)	84.1 (8.8)
Creatinine, mg/dL	0.7 (0.3)	0.71 (0.17)	0.79 (0.18)
Albumin, g/dL	4.4 (0.3)	4.4 (0.3)	4.3 (0.3)
Total proteins, g/dL	7.3 (0.4)	7.2 (0.3)	7.5 (0.5)
Prealbumin, g/dL	22.1 (5.0)	22.4 (4.6)	21.4 (6.1)
$\mathrm{Radiological}\;\mathrm{extension},\;\bar{x}\;($SD)			
Total extension score	8.1 (3.3)	7.1 (3.0)	10.2 (3.0) *
Bronchial dilatation score	1.2 (0.2)	1.2 (0.2)	1.2 (0.2)
Bronchial wall thickness score	1.3 (0.3)	1.3 (0.4)	1.3 (0.3)
Global score	10.6 (3.4)	9.6 (3.2)	12.8 (2.9) *
Treatments			
Bronchodilators, N	23 (77%)	16 (76%)	7 (78%)
Inhaled corticoids, N	10 (33%)	6 (29%)	4 (44%)
Mucolytics, N	2 (7%)	2 (10%)	0
Eradication protocol for PA	9 (30%)	NA	9 (100%)
Respiratory physiotherapy, N	14 (47%)	12 (57%)	2 (22%)

Values are presented as mean (standard deviation). Abbreviations: N, number; BMI, body mass index; kg, kilograms; cm, centimeters; BMI, body mass index; m, meters; COPD, Chronic Obstructive Pulmonary Disease; FEV1, forced expiratory volume in the first second; FVC, forced vital capacity; L, liter; FACED: F, FEV1, forced expiratory volume in the first second; A, Age; C, chronic colonization by *Pseudomonas aeruginosa*; E, radiological extension; D, dyspnea; EFACED: E, exacerbations with hospitalization in previous year; F, FEV1, forced expiratory volume in the first second; A, Age; C, Chronic colonization by *Pseudomonas aeruginosa*; E, radiological extension; D, dyspnea; BSI, Bronchiectasis Severity Index; mMRC, modified medical research council; #, number; g, grams; dL, deciliter; mg, milligrams, PA, *Pseudomonas aeruginosa*, NA, not available. Statistical analyses and significance: *, *p* ≤ 0.05 between *Pseudomonas aeruginosa* colonization patients and non-*Pseudomonas aeruginosa* colonization patients.

**Table 3:** Baseline systemic inflammatory parameters of all bronchiectasis patients, and of the two groups of patients.

$\mathrm{Systemic}\;\mathrm{Inflammatory}\;\mathrm{Parameters},\;\bar{x}\;(\mathrm{SD})$	All Patients N = 30	Non-*Pseudomonas aeruginosa* N = 21	*Pseudomonas* *aeruginosa* N = 9
Total leukocytes, ×10^3^/µL	6.4 (1.6)	6.4 (1.5)	6.3 (1.9)
Total neutrophils, ×10^3^/µL	4.1 (1.4)	4.1 (1.3)	4.0 (1.5)
Neutrophils, %	63.2 (7.8)	63.7 (7.7)	62.0 (8.2)
Total lymphocytes, ×10^3^/µL	1.5 (0.4)	1.5 (0.4)	1.5 (0.4)
Lymphocytes, %	24.5 (6.2)	24.6 (7.0)	24.2 (3.8)
Total eosinophils, ×10^3^/µL	0.16 (0.09)	0.15 (0.07)	0.20 (0.14)
Eosinophils, %	2.5 (1.4)	2.4 (1.1)	2.9 (2.0)
Platelets, ×10^3^/µL	257 (69)	254 (63)	265 (86)
CRP, mg/dL	0.70 (0.9)	0.76 (1.07)	0.60 (0.37)
ESR, mm/h	15 (12)	16 (14)	13 (10)
Fibrinogen, mg/dL	370 (84)	370 (89)	369 (75)
Alpha-1 antitrypsin, mg/dL	132.5 (25.4)	132.8 (26.7)	131.7 (23.7)
Ceruloplasmin, mg/dL	27.0 (5.4)	27.2 (6.3)	26.7 (2.9)
IgE, IU/mL	66 (81)	57 (75)	85 (95)
IgG, mg/dL	1273 (384)	1161 (259)	1535 (506) *
IgG aspergillus, mg/L	37 (35)	37 (37)	36 (30)
IgM, mg/dL	112 (85)	83 (42)	150 (94) *
IgA, mg/dL	330 (134)	295 (120)	390 (152) *

Values are presented as mean (standard deviation). Abbreviations: N, number; CRP, C-reactive protein; ESR, erythrocyte sedimentation rate; IgE, immunoglobulin E; IgG, immunoglobulin G; IgG aspergillus, immunoglobulin G aspergillus; IgM, immunoglobulin M; IgA, immunoglobulin A; IgG, immunoglobulin G; µL, microliter; mg, milligrams; mm, millimeters; h, hour; dL, deciliter; mL, millilitre. Statistical analyses and significance: *, *p* ≤ 0.05 between *Pseudomonas aeruginosa* colonization patients and non-*Pseudomonas aeruginosa* colonization patients.

**Table 4:** Number of exacerbations and hospitalizations for exacerbations in each patient in the previous year.

Patients	# Exacerbations/Patient/Year		# Hospitalizations/Patient for Exacerbations in the Previous Year	
	Baseline	One-Year Follow-Up	Baseline	One-Year Follow-Up
Patient # 1	1	1	0	0
Patient # 2	1	2	0	0
Patient # 3	0	2	0	0
Patient # 4	1	0	1	0
Patient # 5	1	0	0	0
Patient # 6	1	2	0	0
Patient # 7	0	1	0	0
Patient # 8	0	1	0	0
Patient # 9	0	4	0	0
Patient # 10	2	NA	0	NA
Patient # 11	1	2	0	0
Patient # 12	1	2	0	0
Patient # 13	0	0	0	0
Patient # 14	0	3	0	0
Patient # 15	0	0	0	0
Patient # 16	1	1	0	0
Patient # 17	0	2	0	0
Patient # 18	1	0	0	0
Patient # 19	1	1	1	1
Patient # 20	0	1	0	0
Patient # 21	1	1	0	0
Patient # 22	2	0	0	0
Patient # 23	2	3	0	0
Patient # 24	2	NA	0	NA
Patient # 25	1	2	0	1
Patient # 26	1	2	0	0
Patient # 27	3	NA	1	NA
Patient # 28	0	0	0	0
Patient # 29	0	1	0	0
Patient # 30	1	1	0	0

Abbreviations: #, number; NA, not available.

**Table 5:** General characteristics at one-year follow-up in bronchiectasis patients.

$\mathrm{Anthropometry},\;\bar{x}\;(\mathrm{SD})$	Baseline	One-Year Follow-Up
Age, years	66 (12)	67 (12) ***
Female, N/male, N	20/7	20/7
Body weight, kg	65 (17)	65 (17)
Height, cm	159 (10)	159 (10)
BMI (kg/m^2^)	25.4 (4.4)	25.3 (4.5)
Etiology		
Post-infectious, N (%)	20 (74)	20 (74)
COPD, N (%)	0 (0)	0 (0)
Unknown etiology, N (%)	7 (26)	7 (26)
$\mathrm{Lung}\;\mathrm{function}\;\mathrm{and}\;\mathrm{exercise}\;\mathrm{capacity},\;\bar{x}\;($SD)		
FEV_1_, % predicted	76.1 (24.8)	74.4 (25.5)
FVC, L	2.69 (0.95)	2.54 (0.86)
FVC, % predicted	85 (19)	85 (18)
FEV_1_/FVC	68.3 (11.8)	67.4 (12.4)
6-min walking distance, meters	490 (89)	477 (96)
Distance, % predicted	101 (16)	97 (16)
Smoking history		
Ex-smokers, N (%)	7 (26)	7 (26)
Never smokers, N (%)	20 (74)	20 (74)
$\text{Packs-year},\;\bar{x}\;($SD)	23 (16)	23 (16)
Disease severity		
$\mathrm{BSI}\;\mathrm{score},\;\bar{x}\;($SD)	5.2 (2.9)	5.7 (2.1)
$\mathrm{mMRC}\;\mathrm{score},\;\bar{x}\;($SD)	0.67 (0.68)	0.70 (0.78)
$\mathrm{Hospitalizations}\;\mathrm{for}\;\mathrm{exacerbations}\;\mathrm{in}\;\mathrm{the}\;\mathrm{previous}\;\mathrm{year},\;\bar{x}\;($SD)	0.07 (0.27)	0.07 (0.27)
$\mathrm{Nutritional}\;\mathrm{assessment},\;\bar{x}\;($SD)		
Hemoglobin, g/dL	14.0 (1.2)	13.5 (1.0) *
Hematocrit, %	42.0 (3.5)	39.2 (10.4)
Glucose, mg/dL	94.3 (26.3)	97.0 (23.8)
Creatinine, mg/dL	0.74 (0.18)	0.75 (0.15)
Albumin, g/dL	4.4 (0.2)	4.4 (0.3)
Total proteins, g/dL	7.3 (0.3)	7.2 (0.4)
Prealbumin, g/dL	22.3 (4.5)	21.8 (4.6)
$\mathrm{Radiological}\;\mathrm{extension},\;\bar{x}\;($SD)		
Total extension score	8.2 (3.2)	NA
Bronchial dilatation score	1.2 (0.2)	NA
Bronchial wall thickness score	1.3 (0.3)	NA
Global score	10.7 (3.3)	NA

Values are presented as mean (standard deviation). Abbreviations: N, number; BMI, body mass index; kg, kilograms; cm, centimeters; BMI, body mass index; m, meters; COPD, Chronic Obstructive Pulmonary Disease; FEV_1_, forced expiratory volume in the first second; FVC, forced vital capacity; L, liter; FACED: F, FEV_1,_ force expiratory volume in the first second; A, Age; C, chronic colonization by *Pseudomonas aeruginosa*; E, radiological extension; D, dyspnea; EFACED: E, exacerbations with hospitalization in previous year; F, FEV_1,_ forced expiratory volume in the first second; A, Age; C, Chronic colonization by *Pseudomonas aeruginosa*; E, radiological extension; D, dyspnea; BSI, Bronchiectasis Severity Index; mMRC, modified medical research council; g, grams; dL, deciliter; mg, milligrams; NA, not available. Statistical analyses and significance: *, *p* ≤ 0.05, ***, *p* ≤ 0.001 between baseline and one-year follow-up bronchiectasis patients.

**Table 6:** Systemic inflammatory parameters at one-year follow-up in bronchiectasis patients.

$\mathrm{Systemic}\;\mathrm{Inflammatory}\;\mathrm{Parameters},\;\bar{x}\;(\mathrm{SD})$	Baseline	One-Year Follow-Up
Total leukocytes, ×10^3^/µL	6.3 (1.6)	6.2 (1.5)
Total neutrophils, ×10^3^/µL	4.1 (1.4)	4.0 (1.2)
Neutrophils, %	63.0 (8.1)	62.5 (8.1)
Total lymphocytes, ×10^3^/µL	1.5 (0.4)	1.5 (0.5)
Lymphocytes, %	24.6 (6.5)	25.2 (8.6)
Total eosinophils, ×10^3^/µL	0.17 (0.09)	0.16 (0.08)
Eosinophils, %	2.8 (1.3)	3.0 (1.7)
Platelets, ×10^3^/µL	262 (70)	241 (56) *
CRP, mg/dL	0.59 (0.63)	0.51 (0.46)
Fibrinogen, mg/dL	379 (62)	396 (70)
Alpha-1 antitrypsin, mg/dL	130.8 (25.3)	131.3 (22.3)

Values are presented as mean (standard deviation). Abbreviations: N, number; CRP, C-reactive protein; ESR, erythrocyte sedimentation rate; IgE, immunoglobulin E; IgG aspergillus, immunoglobulin G aspergillus; IgM, immunoglobulin M; IgA, immunoglobulin A; IgG, immunoglobulin G; µL, microliter; mg, milligrams; mm, millimeters; h, hour; IU, international unit; dL, deciliter; L, liter. Statistical analyses and significance: *, *p* ≤ 0.05 between baseline and one-year follow-up bronchiectasis patients.

**Table 7:** Sputum and microbiological status at baseline and at one-year follow-up.

Patients	Baseline		One Year Follow-Up	
	Germs	Score	Germs	Score
Patient # 1	*Haemophilus influenza*, S	5	*Haemophilus influenza*, S	5
Patient # 2	*Moraxella catarrhalis*, S	5	NC, S	1
Patient # 3	*Pseudomonas aeruginosa*, S	3	*Pseudomonas aeruginosa*, S	5
Patient # 4	*Pseudomonas aeruginosa*, S	3	*Pseudomonas aeruginosa*, S	5
Patient # 5	Commensal microbiota, S	5	*Moraxella catarrhalis*, S	4
Patient # 6	*Pseudomonas aeruginosa*, S	5	NC, S	2
Patient # 7	Commensal microbiota, S	6	NSA	NA
Patient # 8	*Pseudomonas aeruginosa*, S	5	*Pseudomonas aeruginosa*, S	3
Patient # 9	*Pseudomonas aeruginosa*, S	5	*Pseudomonas aeruginosa*, S	6
Patient # 10	Commensal microbiota, S	5	NA	NA
Patient # 11	Commensal microbiota, S	5	Commensal microbiota, S	4
Patient # 12	Commensal microbiota, S	5	NC, S	1
Patient # 13	NSA, I	NA	NSA	NA
Patient # 14	*Pseudomonas aeruginosa*, S	5	Commensal microbiota, S	4
Patient # 15	Commensal microbiota, S	6	NC, S	1
Patient # 16	*Pseudomonas aeruginosa*, S	5	NSA	NA
Patient # 17	Commensal microbiota, S	5	*Stenotrophomonas maltophilia*	5
Patient # 18	NSA, I	NA	NSA	NA
Patient # 19	Commensal microbiota, S	5	NSA	NA
Patient # 20	Commensal microbiota, S	5	*Haemophilus influenza*, S	4
Patient # 21	Commensal microbiota, S	6	Commensal microbiota, S	3
Patient # 22	NC, S	2	NC, S	1
Patient # 23	Commensal microbiota, S	3	Commensal microbiota, S	4
Patient # 24	NSA, I	NA	NA	NA
Patient # 25	NSA, I	NA	*Pseudomonas aeruginosa*, S	4
Patient # 26	Commensal microbiota, S	6	NC, S	1
Patient # 27	*Pseudomonas aeruginosa*, S	6	NA	NA
Patient # 28	Commensal microbiota, S	6	*Pseudomonas aeruginosa*, S	3
Patient # 29	*Pseudomonas aeruginosa*, S	6	*Pseudomonas aeruginosa*, S	5
Patient # 30	Commensal microbiota, S	6	NSA	NA

Abbreviations: S, spontaneous; I: induced; NSA, no sputum available; NA, not available; NC, no culture.

## Data Availability

The datasets generated and analyzed during the current study are available from the corresponding author on reasonable request.
